# Clinical and molecular characteristics of a novel rare *de novo* variant in *PPP2CA* in a patient with a developmental disorder, autism, and epilepsy

**DOI:** 10.3389/fcell.2022.1059938

**Published:** 2022-11-30

**Authors:** Iris Verbinnen, Sara S. Procknow, Lisa Lenaerts, Sara Reynhout, Aujan Mehregan, Chris Ulens, Veerle Janssens, Katherine A. King

**Affiliations:** ^1^ Laboratory of Protein Phosphorylation and Proteomics, Department of Cellular and Molecular Medicine, University of Leuven (KU Leuven), Leuven, Belgium; ^2^ KU Leuven Brain Institute (LBI), Leuven, Belgium; ^3^ Division of Genetics and Genomic Medicine, Department of Pediatrics, Washington University in St. Louis, St. Louis, MO, United States; ^4^ Laboratory of Structural Neurobiology, Department of Cellular and Molecular Medicine, University of Leuven (KU Leuven), Leuven, Belgium

**Keywords:** PP2A-related neurodevelopmental disorders, *de novo* mutation, PPP2CA, epilepsy, autism (ASD), developmental delay, case report

## Abstract

PP2A-related (neuro) developmental disorders are a family of genetic diseases caused by a heterozygous alteration in one of several genes encoding a subunit of type 2A protein phosphatases. Reported affected genes, so far, are *PPP2R5D*, encoding the PP2A regulatory B56δ subunit; *PPP2R1A*, encoding the scaffolding Aα subunit; and *PPP2CA*, encoding the catalytic Cα subunit—in that order of frequency. Patients with a pathogenic *de novo* mutation in one of these genes, in part, present with overlapping features, such as generalized hypotonia, intellectual and developmental delay, facial dysmorphologies, seizures, and autistic features, and, in part, with opposite features, e.g., smaller versus larger head sizes or normal versus absent corpus callosum. Molecular variant characterization has been consistent so far with loss-of-function or dominant-negative disease mechanisms for all three affected genes. Here, we present a case report of another *PPP2CA*-affected individual with a novel *de novo* missense variant, resulting in a one-amino acid substitution in the Cα subunit: p.Cys196Arg. Biochemical characterization of the variant revealed its pathogenicity, as it appeared severely catalytically impaired, showed mildly affected A subunit binding, and moderately decreased binding to B/B55, B”/PR72, and all B56 subunits, except B56γ1. Carboxy-terminal methylation appeared unaffected, as was binding to B”’/STRN3—all being consistent with a partial loss of function. Clinically, the girl presented with mild-to-moderate developmental delay, a full-scale IQ of 83, mild dysmorphic facial features, tonic–clonic seizures, and autistic behaviors. Brain MRI appeared normal. We conclude that this individual falls within the milder end of the clinical and molecular spectrum of previously reported *PPP2CA* cases.

## 1 Introduction

PP2A-related neurodevelopmental disorders (NDDs) are a group of rare genetic diseases characterized by heterozygous *de novo* mutations in *PPP2CA* (Reynhout et al., 2019) (OMIM: #618354), *PPP2R1A* (Houge et al., 2015; Lenaerts et al., 2020; Douzgou et al., 2022) (OMIM: #616362), or *PPP2R5D* (Houge et al., 2015; Loveday et al., 2015; Shang et al., 2016; Mirzaa et al., 2019; Oyama et al., 2022) (OMIM: #616355). These three PP2A genes belong to nineteen human genes encoding the protein phosphatase 2A (PP2A) family of Ser/Thr phosphatases (Janssens and Goris, 2001; Lambrecht et al., 2013), a group of dephosphorylating enzymes with pleiotropic functions in cell signaling and organismal physiology (Janssens and Goris, 2001; Reynhout and Janssens, 2019). Specifically, in the brain, PP2A phosphatases regulate cortical development, synaptic transmission, hippocampus-dependent memory, dopaminergic signaling, and tau phosphorylation (Sandal et al., 2021; Verbinnen et al., 2021). Structurally, PP2A phosphatases comprise at least two subunits: a catalytic C and a scaffolding A subunit, which, in the majority of cases, additionally bind to a third, regulatory B subunit. Through these B subunits, trimeric PP2A complexes achieve their substrate specificity, regulation, subcellular localization, and tissue-specific expression (Slupe et al., 2011; Lambrecht et al., 2013). Despite this major structural complexity, PP2A-related NDDs are characterized by alterations in just a subset of PP2A holoenzymes, and the molecular characterization of *PPP2CA* (encoding the catalytic Cα subunit), *PPP2R1A* (encoding the scaffolding Aα subunit), and *PPP2R5D* (encoding the regulatory B56δ subunit) variants have been consistent so far with a loss-of-function mechanism for most, if not, all of them (Houge et al., 2015; Reynhout et al., 2019; Lenaerts et al., 2020; Oyama et al., 2022). Although PP2A affected individuals exhibit common clinical features, including hypotonia, developmental delay (DD) (motoric skills, speech), intellectual disability (ID), differences in brain size, autism (ASD), and seizures, they also show a broad heterogeneity in the severity of their presentation—within individuals affected in different PP2A genes, as well as within individuals affected in the same PP2A gene. In addition, the frequency by which a PP2A gene alteration occurs within the general population is significantly different between genes, with most cases so far discovered for *PPP2R5D* and *PPP2R1A*.

Here, we describe a new case of a currently 18-year-old girl with a developmental disorder, autistic features, epilepsy, and a novel, *de novo* pathogenic variant of *PPP2CA*. Based on 16 previously published individuals, the *PPP2CA*-related disorder has a rather heterogeneous clinical presentation, with mildly to severely affected individuals, and with 15 different reported pathogenic variants dispersed throughout the protein or the gene (Reynhout et al., 2019). Based on the clinical and molecular data described below, we find that this new case falls well within the spectrum of previously reported *PPP2CA* cases.

## 2 Materials and methods

### 2.1 Generation of the *PPP2CA* p.Cys196Arg variant

The coding region of wild-type (WT) Cα complementary DNA (cDNA) was cloned into an N-terminal HA-tag eukaryotic expression vector (pMB001) using *Xba*I/*Bam*HI sites. The mutated *PPP2CA* construct was directly generated from this plasmid by polymerase chain reaction (PCR)-based site-directed mutagenesis (Stratagene) with *Pwo* polymerase (Roche Applied Science) and oligonucleotides (IDT) containing the desired point mutations. Forward and reverse primer sequences were 5′-CCA​TGA​GGG​TCC​AAT​GCG​TGA​CTT​GCT​GTG​GTC-3′ and 5′-GAC​CAC​AGC​AAG​TCA​CGC​ATT​GGA​CCC​TCA​TGG-3′, respectively. Introduction of the variant was verified by Sanger sequencing (LGC Genomics).

### 2.2 Cellular PP2A binding assays

HEK293T cells (ATCC, characterized by short tandem repeat profiling and mycoplasma-free) were transfected with PEI or PEI MAX transfection reagent using the standard protocols. All GFP-B-type subunit expression vectors have previously been described (Janssens et al., 2003; Houge et al., 2015; Haesen et al., 2016). The GFP expression plasmid, pEGFP-C1, was from Clontech. Seventy-two hours post-transfection, cells were rinsed with phosphate buffered saline (PBS), lysed in 150 μl NET buffer (50 mM Tris.HCl pH 7.4, 150 mM NaCl, 15 mM EDTA, and 1% Nonidet P-40) containing protease and phosphatase inhibitor cocktail (Roche Applied Science), and centrifuged for 15 min at 13,000 *g*. For binding assays with GFP-STRN3, no phosphatase inhibitors were added. If the experiment required the measurement of phosphatase activity, Tris buffered saline (TBS) was used instead of PBS, and phosphatase inhibitors were omitted from the lysis buffer.

For pulldown experiments, lysates were incubated at 4°C for 1 h with 700 µl NENT100 buffer (20 mM Tris.HCl pH 7.4, 1 mM EDTA, 0.1% Nonidet P-40, 25% glycerol, 100 mM NaCl) containing 1 mg/ml bovine serum albumin and 30 μl anti-HA-Agarose beads (Sigma-Aldrich, for HA pull-down) or 30 μl GFP-trap-A beads (ChromoTek, for GFP pulldown). Beads were washed three times with 1 ml NENT300 (containing 300 mM NaCl) and two times with 1 ml NENT150 (containing 150 mM NaCl). Bound proteins were eluted in 2× NuPage sample buffer (Invitrogen) and boiled for subsequent analysis by sodium dodecyl sulfate-polyacrylamide gel electrophoresis (SDS-PAGE) on 4–12% (w/v) Bis-Tris gels (Thermo Fisher Scientific) and Western blotting. Membranes were blocked in 5% milk in TBS/0.1% Tween-20 for 1 h at room temperature and incubated with the primary antibody overnight at 4°C. Primary mouse monoclonal antibodies were anti-HA (clone HA-7, Sigma-Aldrich), anti-GFP (B-2) (sc-9996), anti-PP2A-A subunit (clone C5.3D10, generously supplied by Dr. S. Dilworth, Middlesex University, London, UK), anti-PP2A-demethylated C subunit (clone 4b7, Merck-Millipore), and anti-vinculin (clone hVIN-1, Sigma-Aldrich). After washing in TBS/0.1% Tween-20, the membranes were incubated with horseradish peroxidase-conjugated secondary antibodies (Dako for anti-mouse and Cell Signaling for anti-rabbit) and developed using Western Bright ECL (Advansta) on the ImageQuant LAS4000 scanner (GE Healthcare). All densitometric quantifications were performed with Image Studio Lite software (version 5.2).

### 2.3 PP2A activity assays

After HA pull-down, beads were washed once more with 20 mM Tris HCl (pH 8.0) and 1 mM DTT (Tris-DTT) and finally resuspended in 80 µl Tris-DTT solution. All assays were performed with 35 µl of this phosphatase suspension and 10 µl of 2 mM stock of K-R-pT-I-R-R phospho-peptide for 10–20 min at 30°C (still in the linear range of the assay). The released free phosphate was determined by the addition of BIOMOL Green (catalog no. BMLAK111-0250, Enzo). After 30 min of incubation at RT, absorbance at 620 nm was measured in a multi-channel spectrophotometer. We subsequently obtained specific phosphatase activity by correcting the measured absorbance for the input of HA-tagged Cα, as determined by immunoblotting with anti-HA antibodies and signal quantification by Image Studio Lite software (version 5.2).

### 2.4 Statistics

Statistical analysis of biochemical data was assessed with unpaired Student’s *t-*test or with one-sample Student’s *t-*test in which the data were compared with WT values that were set at 100% in each experimental replicate. *p* values below 0.05 were considered significant.

### 2.5 Protein stability prediction and variant modeling

Protein stabilities were calculated using the FoldX force field (Schymkowitz et al., 2005). For details of the FoldX calculations, see the reference. The force field was run in a command line interface. In brief, the crystal structure of *PPP2CA*, as determined within the PP2A-B56γ heterotrimer (Cho and Xu, 2007), was used as the input for the initial refinement step performed by the RepairPDB command in FoldX. This produced an energy-minimized PDB file where the BuildModel command could then be used to incorporate the Cys196Arg substitution while keeping steric hindrances and energies at a minimum. The output with the incorporated Arg196 substitution was then used for the AnalyseComplex command. This command ran *n* = 3 calculations and generated overall ΔG values associated with the WT and variant models, so that ΔΔG values could be calculated.

AlphaFold was used to predict the structure of the *PPP2CA* p.Cys196Arg mutant. The open source Google Colab version of AlphaFold2 (ColabFold) was used for model prediction (Mirdita et al., 2022). The output model from AlphaFold was superimposed on the crystal structure of WT *PPP2CA* (PDB ID: 2IAE) for analysis in PyMOL (root mean square deviation: 0.511).

## 3 Results

### 3.1 Clinical findings

The patient was the full-term product of an uncomplicated pregnancy. The birth weight and length were within normal limits (weight z = −0.25 and length z = 0.89 per WHO Girls 0–2 growth charts). There were early concerns for poor breast-feeding and failure to gain appropriate weight that resolved after changing to formula. The patient had torticollis in infancy that resolved with physical therapy and required a helmet for the resulting positional plagiocephaly. There were no other concerns in the neonatal period.

She had two unprovoked generalized tonic–clonic seizures at age 1.5 years and two additional generalized tonic–clonic seizures at age 3, associated with fever. She was placed on Tegretol from age 2–5 and remained seizure free until age 12 when she had a recurrence of tonic–clonic seizures. The seizures continued to increase in frequency despite medications, although she had required medication changes due to side effects, including excessive sedation and anxiety. The brain MRI completed at age 12 was normal.

There were concerns for developmental delay beginning at 6 months as the patient was not achieving developmental milestones as quickly as an older sibling, but early milestones were within normal limits. The patient sat independently at approximately 6 months and stood at 10–11 months. She was walking by 14 months of age. She said her first word at 12 months of age, but she communicated through pointing/grunting until she started using sentences at age 3.5 years. She toilet trained at 3.5 years but continued to have nocturnal bed wetting until age 9. Academic difficulty was noted early, with particular difficulty with speech, articulation, and reading. She was initially in a common classroom environment but eventually required full-time special education assistance. At age 11, full-scale IQ testing showed an IQ of 83. Developmental testing at that time led to the diagnosis of autism, and she was noted to have extremely low adaptive functioning. She is currently 18 years old and reads at a 5^th^-grade level with difficulty. She has difficulty with social interactions, has restrictive and repetitive behaviors, and is hyper- or hypo-reactive to sensory input. She developed selective mutism and significant anxiety as a child (<10 years of age), requiring medication, as well as skin-picking behaviors. She also has a history of significant textural food aversion developing around the same age leading to hospitalization at age 16 for malnutrition in the setting of avoidant restrictive food intake disorder. She had improvement in weight gain with Mirtazapine. The patient is also nearsighted, while her hearing ability is within normal limits. At the gastrointestinal level, she suffers from constipation and GERD.

### 3.2 Family history

**FIGURE 1:**
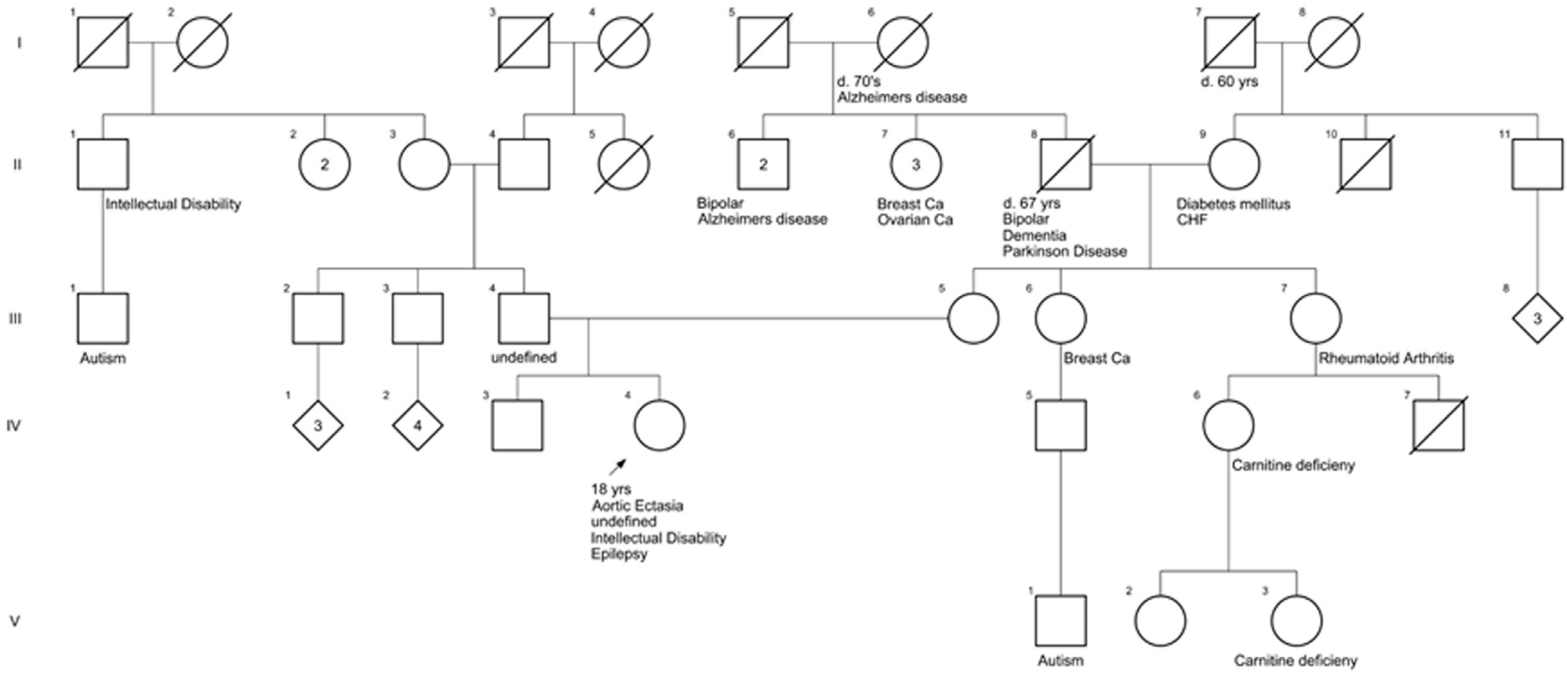
Family history of the patient. The pedigree shows pathologic features of family members closely related to the patient.

To reveal a potential familial cause, the patient’s family history was collected. The patient was of northern European ancestry. Her father (III-4) has a mildly enlarged aorta, but the immediate family is otherwise healthy (Figure 1). There is a distant paternal granduncle with suspected intellectual disability (II-1), and his son (III-1) is suspected to have autism. Maternal grandfather (II-8) had bipolar disorder and Lewy body dementia vs. Parkinson’s disease when he died at age 67. There is a maternal granduncle (II-6) with bipolar disorder and a maternal granduncle (II-6) with Alzheimer’s disease, as did the patient’s maternal great-grandmother (I-6). There is a maternal first cousin (IV-6) and first cousin once removed (V-3) with L-carnitine deficiency. There is a maternal first cousin with suspected autism (V-1) (Figure 1).

### 3.3 Genetics

**FIGURE 2:**
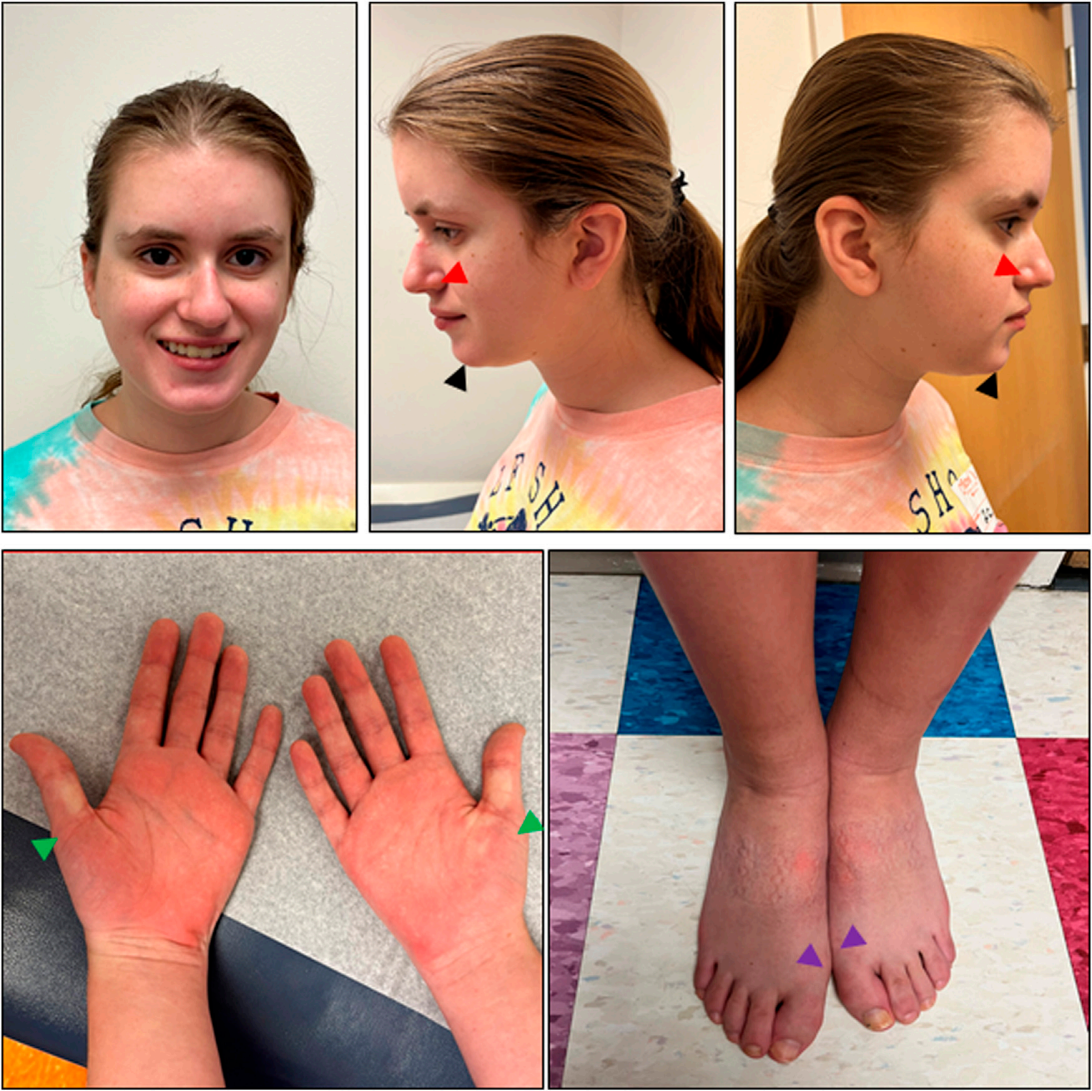
Photographs of the patient. Facial features of this patient (age 18 years, 1 month) include a long face, deep set eyes, thin alae nasi (red arrows) with prominent nasal tip, and wide mouth with prominent chin (black arrows). Bilateral hands have proximally placed thumbs (green arrows), and bilateral feet have broad hallices (purple arrows). At the time that photographs were taken, the patient had a fungal nail infection that was healing. She also had marks of her socks on her feet. Informed consent was provided by the parents.

The patient was first evaluated by Medical Genetics at age 10 due to a tall, thin body habitus and re-evaluated at age 16 due to autism and epilepsy. At the most recent evaluation, she was noted to have a slight build (weight z = −0.86, height z = −0.3) with relative microcephaly (z = −1.48 Nellhaus Girls). The exam was significant for a thin body habitus as well as a long face, deep-set eyes, thin alae nasi with prominent nasal tip, and wide mouth with a prominent chin (Figure 2). She had mild limitation of elbow extension and bilateral mild pes planovalgus. Both hands had proximally placed thumbs, and the feet had broad hallices bilaterally (Figure 2). A neurologic exam showed normal cranial nerves, normal tone (no hypotonia), normal reflexes, and normal coordination/gait. The echocardiogram showed a mildly dilated ascending aorta, likely secondary to low body surface area (2.64 cm, z = 2.4). The workup included normal ammonia and chromosomal microarray. The karyotype revealed a chromosome 9p12q13 inversion thought to be benign. An extensive autism/intellectual disability gene sequencing panel was negative. She subsequently underwent trio clinical exome and mitochondrial DNA sequencing that revealed a *de novo* heterozygous variant in *PPP2CA* (c.586T>C, p.Cys196Arg), as well as biallelic variants in *SKIV2L* [paternally inherited c.2932G>A (p.G978R) and maternally inherited c.3254G>A (p.S1085N)].

Pathogenic variants in *SKIV2L* are associated with severe, intractable diarrhea with onset in the first few weeks of life (OMIM: #614602, reviewed in Fabre et al., 2012). While the patient had compound heterozygous variants in the *SKIV2L* gene that are classified as variants of uncertain significance by ACMG criteria (Richards et al., 2015), the patient had no history of intractable diarrhea. Variants in *SKIV2L* are not associated with autism, intellectual disability, seizures, or the patient’s facial or musculoskeletal features. These variants were therefore considered unlikely to contribute to her phenotype.

**FIGURE 3:**
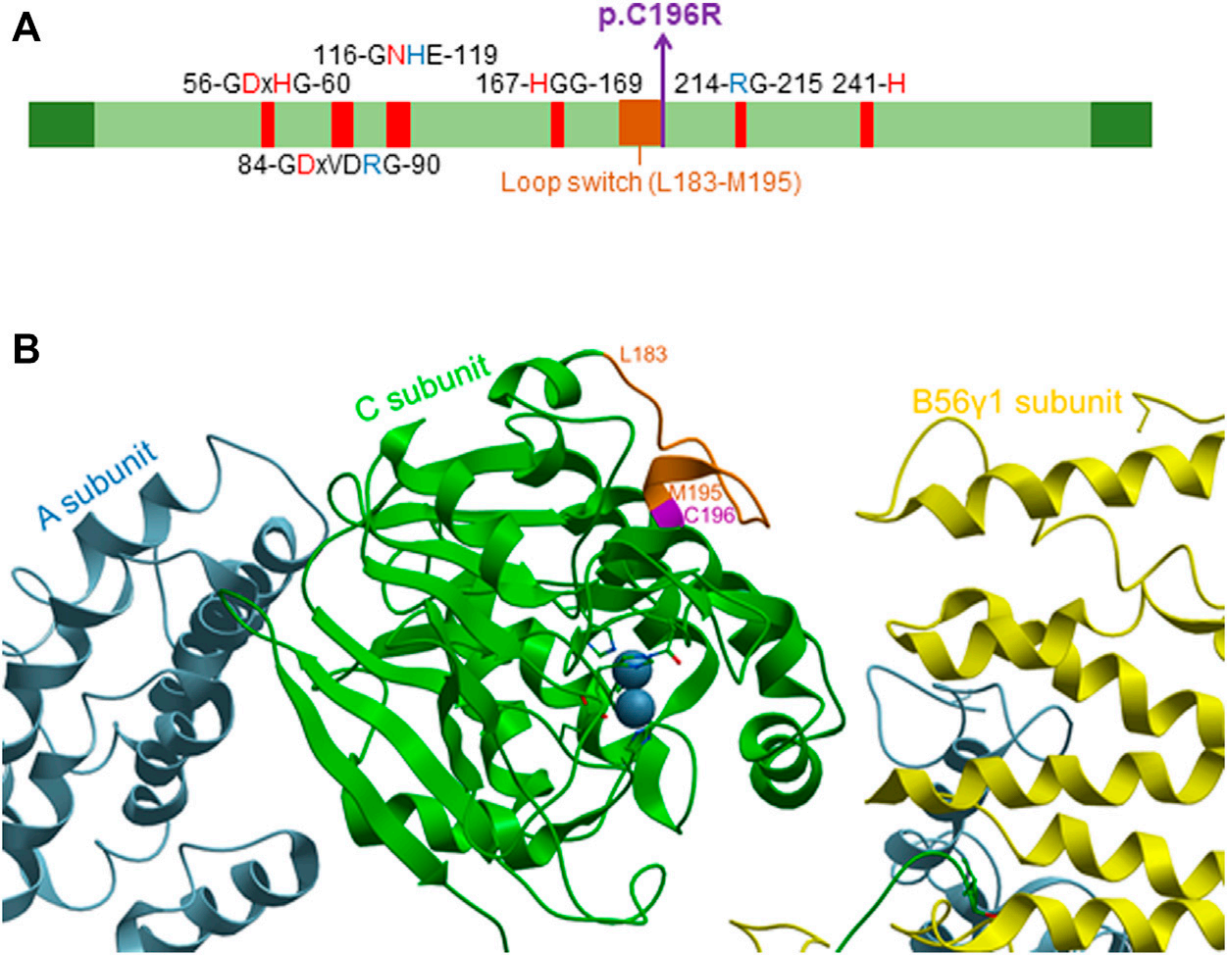
Localization of the p.Cys196Arg variant in the catalytic PP2A Cα subunit. **(A)** Domain organization of the catalytic PP2A Cα subunit. The p.Cys196Arg variant in PP2A Cα is indicated in purple (bold), and other conserved residues in the catalytic subunit are also shown. Red amino acids denote metal binding, while blue amino acids denote phosphate binding. The loop switch is indicated in orange [figure adapted from Vaneynde et al. (2022)]. **(B)** Structure of the catalytic PP2A Cα subunit. The structure was generated based on PP2A-B56γ1 crystallographic data, using PDB code 2IAE (Cho and Xu, 2007) and Molsoft MolBrowser 3.9-2b software (ICM-Browser-Pro). The catalytic Cα subunit is shown in green, the A subunit in sky-blue, and the B56γ1 subunit in yellow. The p.Cys196Arg residue is indicated in purple, while the residues of the loop switch are colored orange.

In contrast, the *PPP2CA* p.Cys196Arg variant was predicted to be disease-causing by MutationTaster (Schwarz et al., 2014). This may be explained by the central position of the mutation in the catalytic core domain of the protein (Figure 3A), and hence, different enzyme features might be affected, including the formation and stabilization of secondary structures (helices and strands) and metal binding (Cho and Xu, 2007). Moreover, the cysteine residue at position 196 is positioned immediately adjacent to the loop switch (amino acids 183–195), which is known to determine the conformation of the active site and the binding of catalytic metal ions (Jiang et al., 2013) (Figures 3A,B). This prompted us to further analyze the biochemical properties of this variant in more detail.

### 3.4 Molecular findings

For functional studies, wild-type Cα (Cα WT) or the Cα p.Cys196Arg variant were ectopically expressed as N-terminally HA-tagged fusion proteins in human embryonic kidney cells (HEK293T).

**FIGURE 4:**
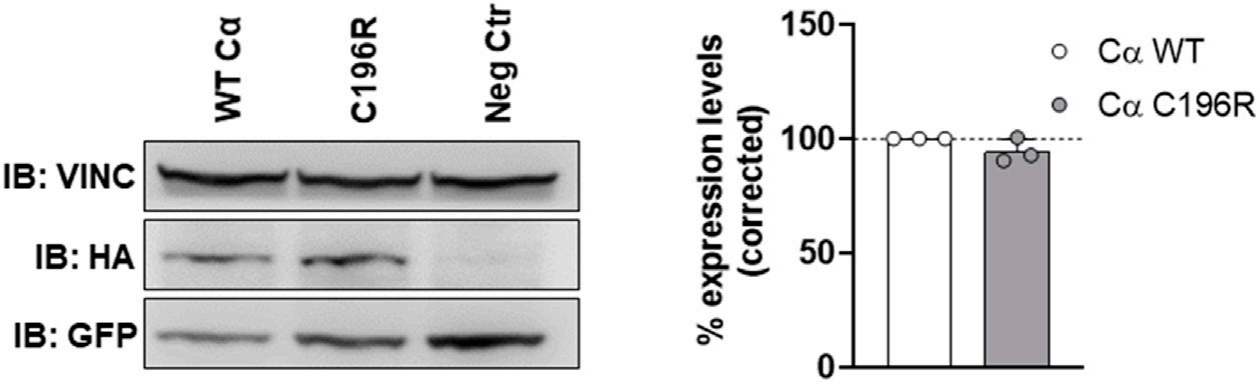
Assessment of variant expression in HEK293T cells. Equal amounts of HA-tagged WT and variant Cα expression plasmids were co-transfected with an equal amount of GFP (green fluorescent protein) expression plasmid. Presence of HA-tagged Cα, GFP and vinculin in the lysates was assessed by anti-HA, anti-GFP, and anti-vinculin immunoblotting, respectively. Left panel: immunoblot from a representative experiment. As a negative control (Neg Ctr), pMB001 vector without insert was co-transfected with a GFP expression plasmid. Right panel: graph displaying expression levels of HA-tagged Cα WT or Cα p.Cys196Arg corrected to an internal (vinculin expression) and transfection efficiency (GFP expression) control. Results represent the average value ±SD of the ratios of the corrected HA signal to the corrected GFP signal in relation to the values of WT Cα (set at 100% in each experiment, dotted line). An unpaired *t* test was used for analyzing statistical significance (*n* = 3).

To determine potential expression issues of the variant, equal amounts of HA-tagged WT and variant Cα expression plasmids were co-transfected with an equal amount of GFP (green fluorescent protein) expression plasmid, allowing us to determine WT and variant expression levels relative to an internal (vinculin expression) and transfection efficiency control (GFP expression). Immunoblot analysis of total protein lysates of transfected cells with anti-HA, anti-vinculin, and anti-GFP antibodies consistently showed comparable expression of Cα WT and Cα p.Cys196Arg (Figure 4).

Next, HA-tagged proteins were isolated from transfected cell lysates on anti-HA-agarose beads, and subjected to three assays (Reynhout et al., 2019): 1) binding to endogenous PP2A A subunit; 2) measurement of catalytic activity; and 3) determination of carboxy-terminal methylation—a well-established post-translational modification of Cα that determines its ability to form (specific) holoenzyme complexes (Janssens et al., 2008; Sents et al., 2013).

**FIGURE 5:**
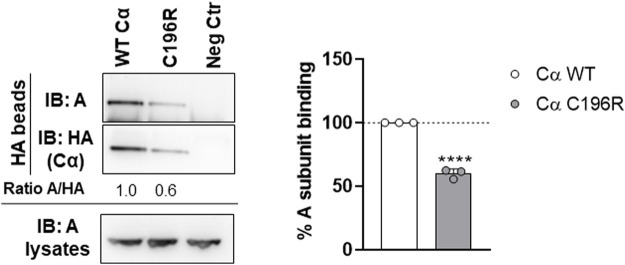
Binding of PP2A Cα p.Cys196Arg to the structural A subunit. HA-tagged Cα WT and p.Cys196Arg proteins were purified from transfected HEK293T cells by HA-pulldown. Presence of endogenous A subunit in the complexes was measured by anti-HA immunoblotting. Left panel: immunoblot from a representative experiment. As a negative control (Neg Ctr) pMB001 vector without insert (expressing only HA tag) was used. Right panel: graph displaying quantified values of A subunit binding. Results represent the average value ±SD of the ratios of the quantified anti-A signal to the quantified anti-HA signal in relation to those values of Cα WT (set at 100% in each experiment, dotted line). An unpaired *t* test was used for analyzing statistical significance (*n* = 3; ****, *p* ≤ 0.0001).

**FIGURE 6:**
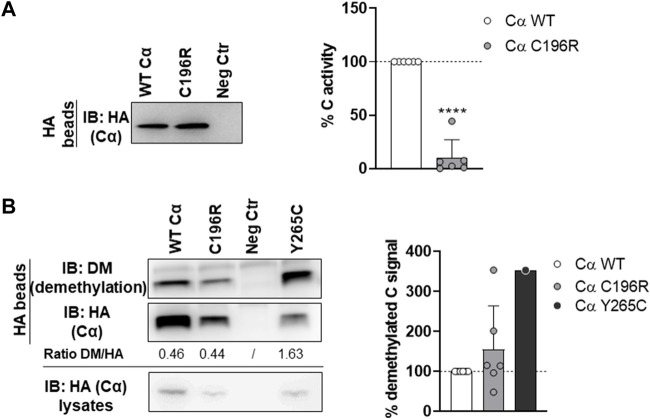
Phosphatase activity and carboxy‐methylation of PP2A Cα p.Cys196Arg. **(A)** Phosphatase activity of the PP2A Cα p.Cys196Arg subunit. HA-tagged Cα WT and p.Cys196Arg proteins were isolated from transfected HEK293T cells by HA-pulldown. PP2A activity was measured on the K-R-pT-I-R-R phospho-peptide using BIOMOL Green. Specific PP2A activity was calculated *via* correction of the measured activities for the Cα inputs (anti-HA immunoblotting). Left panel: immunoblot data from a representative experiment. As a negative control (Neg Ctr) pMB001 vector without insert (expressing only HA tag) was used. Right panel: graph representing the average specific activity ±SD for the Cα p.Cys196Arg variant in comparison to WT activity (set at 100% in each replicate, dotted line). An unpaired *t* test was used for statistical analysis (*n* = 6; ****, *p* ≤ 0.001). **(B)** Carboxy‐methylation status of PP2A Cα p.Cys196Arg. HA-tagged Cα WT and p.Cys196Arg proteins were purified from transfected HEK293T cells by HA-pulldown. PP2A C subunit methylation was assessed by immunoblotting with a demethyl-specific anti-C monoclonal antibody. PP2A Cα p.Tyr265Cys was used as a positive control for low carboxy‐methylation status (hence, high demethyl) (Reynhout et al., 2019). Left panel: immunoblot from a representative experiment. As a negative control (Neg Ctr) pMB001 vector without insert (expressing only HA tag) was used. Right panel: graph displaying the average ratios of the quantified anti-demethyl signals over the quantified anti-HA signals ±SD in comparison to the demethylation levels of Cα WT (set at 100% in each experiment, dotted line). An unpaired *t* test was used for statistical analysis (*n* = 6, except p.Tyr265Cys: *n* = 1).

First, the presence of the A subunit in complex with HA-tagged Cα WT or p.Cys196Arg was analyzed by anti-A immunoblotting. Binding of the structural A subunit to the Cα p.Cys196Arg variant was found moderately impaired compared to Cα WT (Figure 5). Second, the intrinsic catalytic activity of both proteins was determined by incubating the anti-HA beads in complex with Cα WT or p.Cys196Arg with a generic phospho-peptide substrate (K-R-pT-I-R-R) for 10–20 min at 30°C and measuring the release of free phosphate by BIOMOL Green. Results indicated that PP2A activity of the p.Cys196Arg variant was significantly decreased by >90% compared to WT (Figure 6A). Third, we assessed potential changes in carboxy‐methylation of the Cα p.Cys196Arg variant by using commercially available anti-demethyl antibodies. HA-tagged Cα p.Tyr265Cys was used as a positive control of low methylation status (or: high demethylation) (Reynhout et al., 2019). Anti-demethyl immunoblots showed that carboxy-methylation levels of Cα p.Cys196Arg were similar to Cα WT, indicating that Cα p.Cys196Arg could still be properly methylated (Figure 6B). Taken together, we identified Cα p.Cys196Arg as a mutant with moderately decreased A subunit binding, severely compromised catalytic activity, and unaltered carboxy-methylation.

**FIGURE 7:**
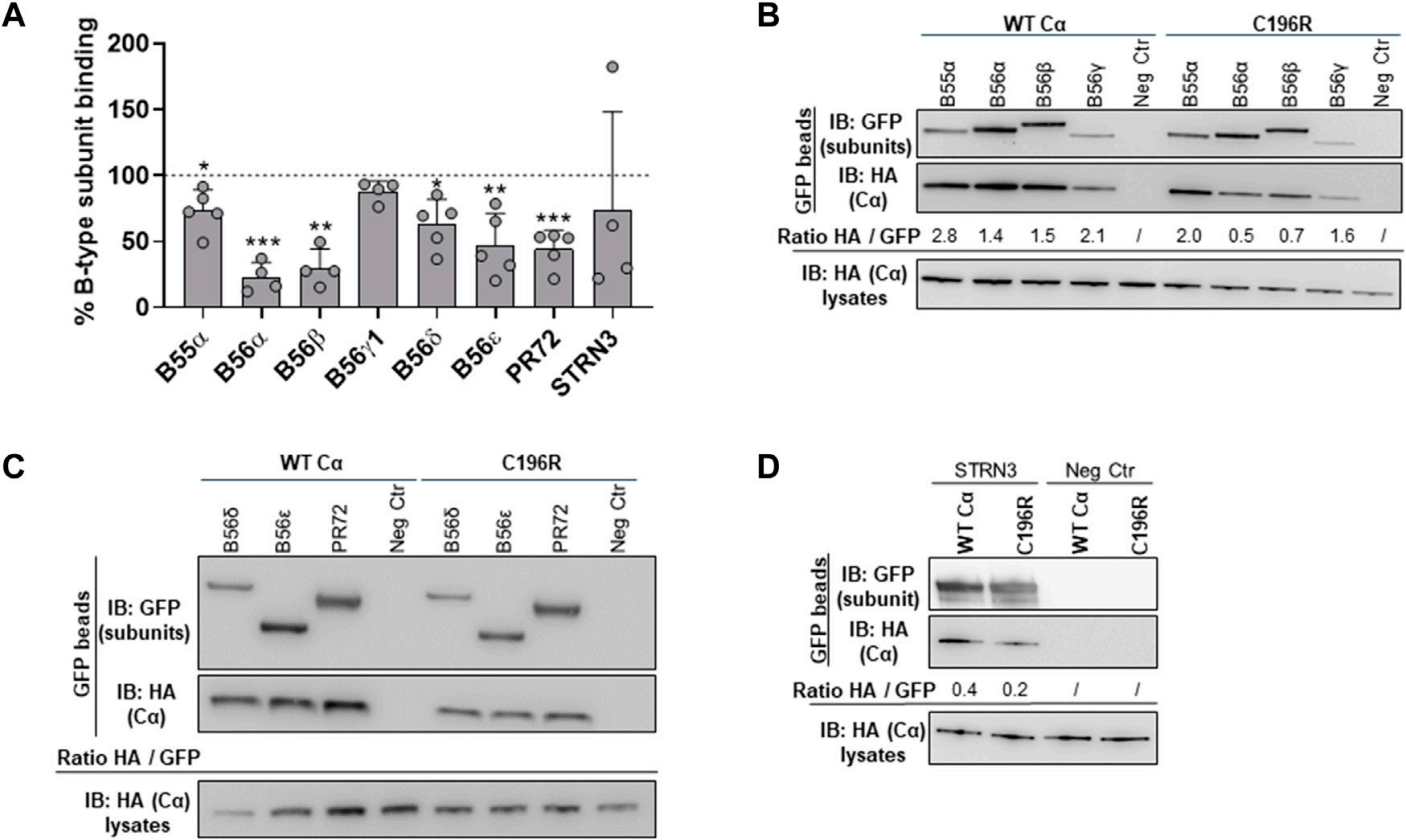
Binding of the PP2A Cα p.Cys196Arg variant to a panel of regulatory B-type subunits. Indicated GFP-tagged B-type subunits were co-expressed with HA-tagged Cα WT or Cα p.Cys196Arg proteins in HEK293T cells. Binding of the variant or Cα WT was assessed in GFP pulldowns by anti-HA immunoblotting. **(A)** Graph showing quantified values of B-type subunit binding. Each bar represents the average value ±SD of the ratios of quantified anti-HA signal to quantified anti-GFP signal for the Cα p.Cys196Arg variant in comparison to Cα WT (set at 100% in each experiment, dotted line). A one-sample *t*-test (compare to 100%) was used for statistical analysis (n ≥ 4; *, *p* ≤ 0.05; **, *p* ≤ 0.01; ***, *p* ≤ 0.001). **(B–D)** Representative blots of binding assays. As a negative control (Neg Ctr), Cα WT or Cα p.Cys196Arg was co-expressed with the pEGFP-C1 vector without insert (only GFP expression).

In a second experimental setup, the HA-tagged Cα WT and mutant cDNAs were co-expressed with several GFP-tagged PP2A B-type subunits in HEK293T cells to assess potential B subunit binding defects (Reynhout et al., 2019). The binding of Cα proteins in the GFP pulldowns was monitored by anti-HA immunoblotting. Although overall binding changes appeared mild, a diverse B-type subunit binding pattern was observed for Cα p.Cys196Arg, characterized by retained binding to B56γ1 and STRN3, whereas binding to B55α, B56α, B56β, B56δ, B56ε, and PR72 was moderately, but statistically significantly decreased, compared to Cα WT (Figures 7A–D).

Thus, biochemical characterization of Cα p.Cys196Arg confirmed its pathogenicity, underscoring that the disease is most likely caused by a functional loss of PP2A Cα enzymatic activity, and being consistent with a loss-of-function pathogenic mechanism.

## 4 Discussion

First reported in 2015–2019 (Fitzgerald et al., 2015; Houge et al., 2015; Reynhout et al., 2019), PP2A-related neurodevelopmental disorders (NDDs) represent a family of rare (neuro)developmental disorders, of which the clinical and molecular spectrum is still likely incomplete, and new diagnoses and variants are likely to further emerge in the near future. Here, we described a case of an 18-year-old girl with a novel *de novo* pathogenic *PPP2CA* variant, which almost certainly explains her clinical features.

So far, only 16 *PPP2CA*-affected individuals have been reported in literature (Reynhout et al., 2019), making *PPP2CA*, by far, the least frequently affected gene of the PP2A family. Based on that single publication, *PPP2CA*-affected cases show a rather heterogeneous clinical presentation, characterized by mild to profound ID and/or DD (100%), seizures (63%), brain abnormalities (67%), hypotonia (69%), ASD or other major behavior problems (47%), and mild to no facial or other dysmorphisms (Reynhout et al., 2019). The female teenager we report here fell well within this spectrum, albeit, clearly, at the milder end. She indeed presented with mild developmental delay, but higher intellectual functioning (IQ of 83 at age 11 years), had normal muscle tone, no brain abnormalities (MRI at age 12 years), and very mild dysmorphic features (Figure 2). On the other hand, she experienced tonic–clonic seizures already at age 1.5 years, and since age 12, these seizures continued to increase despite anti-epileptic medication. In addition, she had difficulty with social interactions, showed restrictive and repetitive behaviors, and was hyper- or hypo-reactive to sensory input, suggestive of autism spectrum disorder. She also developed selective mutism and significant anxiety as a child, as well as avoidant restrictive food intake, both of which required medication.

**FIGURE 8:**
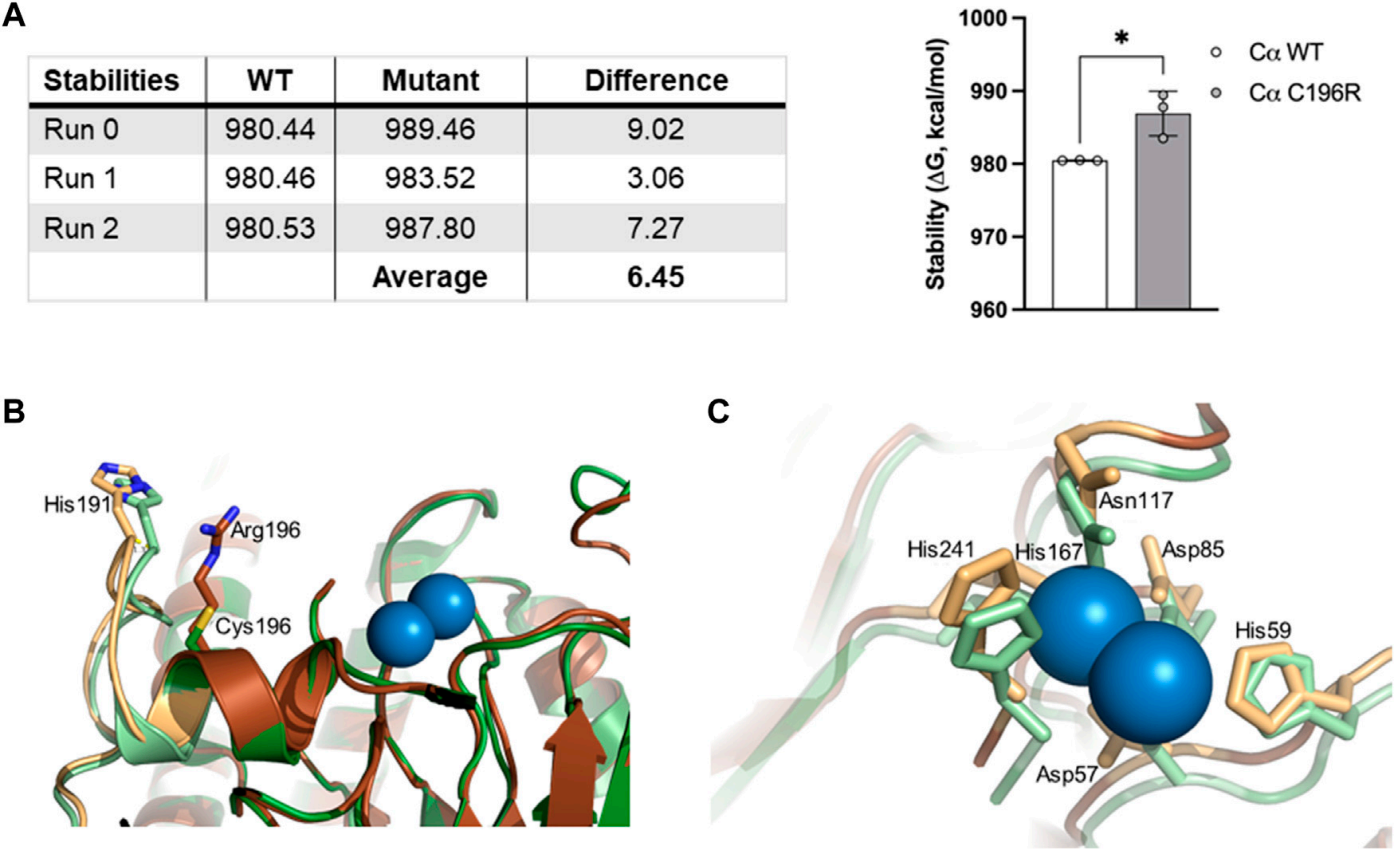
Predicted models for changes in stability and structure of the Cα p.Cys196Arg variant. **(A)** Changes in protein stability between *PPP2CA* WT and p.Cys196Arg variant were predicted by the FoldX algorithm, based on crystallographic data by Cho and Xu (2007) (PDB ID: 2IAE). ΔG values (in kcal/mol) for WT or variant proteins are displayed in the table (obtained in three different runs). The average difference in ΔG between both proteins (ΔΔG) is shown in the graph (^*^, *p* < 0.05). Values in ΔΔG above 0.5 kcal/mol are considered significant. **(B,C)** AlphaFold2-predicted structural changes in the Cα p.Cys196Arg variant (in brown) compared to WT (in green) at the interface with His191 in the loop switch (panel B) and within the catalytic site (panel C). Catalytic metal ions (in blue) and metal coordinating residues (Asp57, His59, Asp85, Asn117, His167, and His241) are indicated. The output model from AlphaFold2 was superimposed on the crystal structure of WT *PPP2CA* (PDB ID: 2IAE) for analysis in PyMOL (root mean square deviation: 0.511).

The biochemical characterization of her *de novo PPP2CA* variant, p.Cys196Arg, concurred with the rather moderate phenotype, as essentially, only a major intrinsic inhibition of PP2A catalytic activity was found, while PP2A methylation was not changed, and complex formation with other PP2A subunits was not (B”/STRN3, B56γ1) or barely affected (40% decrease for A, 25% decrease for B55α, 80% decrease for B56α, 75% decrease for B56β, 40% decrease for B56δ, and 50% decrease for B56ε and B”/PR72). Cys196 is localized near the catalytic center of the C subunit, immediately adjacent to the loop switch (Jiang et al., 2013) (Figures 3A,B). We, thus, suspect that the substitution of the cysteine residue by an arginine affects the conformation of this loop, and consequently, the conformation of the active site and/or binding of catalytic metal ions (Jiang et al., 2013). This conformational change may also further explain the slight decrease in A subunit binding (Jiang et al., 2013), observed for the variant (Figure 5). To further underscore these hypotheses, we used two structure prediction algorithms: FoldX (Schymkowitz et al., 2005) and AlphaFold (Jumper et al., 2021). FoldX force field calculations to determine changes in the stability of Cα p.Cys196Arg revealed that the incorporation of Arg at this position results in a positive ΔG shift of 6.45 ± 3.06 kcal/mol, suggesting an increased destabilization of the protein compared to WT (Figure 8A). Using AlphaFold modeling, we observed that residues 185–194 corresponding to the loop switch were pushed farther from the active site. The largest shift involved His191 (1.3 Å), most likely due to a charge repulsion from Arg196 (Figure 8B). Moreover, we observed that the active site formed by residue side chains of Asp57, His59, Asp85, Asn117, and His241, was larger in the Cα p.Cys196Arg variant (Figure 8C). Based on the AlphaFold prediction, this conformational change is due to Asp57 bending away, along with shifts in His118 and His241 (Figure 8C). Thus, a more open active site with residues such as His59, His118, and His241 pushed astray may adversely affect the proper binding of catalytic metal ions, and thereby impair catalytic activity, as observed in our phosphatase activity assays (Figure 6A). Alternatively, but less likely, Cys196 might be one of ten Cys residues in the Cα subunit that contribute to increased PP2A activity under reducing conditions (Foley and Kintner, 2005; Foley et al., 2007), and hence, its substitution into arginine may in part disturb that regulation. However, unlike other reported pathogenic *PPP2CA* variants with significantly impaired catalytic activity (p.Asp88Gly, p.Tyr127Cys and p.Tyr265Cys) (Reynhout et al., 2019), Carboxy‐methylation of p.Cys196Arg appeared largely normal (Figure 6B), consistent with the observed lack of any major B-type subunit binding defects (Figure 7). Therefore, the molecular profile of the p.Cys196Arg variant we found here perhaps best resembles that of the only reported recurrent *PPP2CA* variant so far, p.His191Arg, which resides in the middle of the loop switch (Jiang et al., 2013; Reynhout et al., 2019) (Figure 3A). The p.His191Arg variant indeed showed decreased catalytic activity, unaffected Carboxy‐methylation, and largely unaffected A- and B-type subunit binding, except for B56δ (severely decreased binding, 14%) and B”’/STRN3 (increased binding, 651%) (Reynhout et al., 2019).

Thus, we should conclude that the observed pattern of biochemical impairments of the p.Cys196Arg variant appears rather unique amongst the currently reported *PPP2CA* variants, but concurring very well with the moderately affected clinical profile of the patient. Although the family pedigree of the girl did show some relatives with intellectual disability or autism, we can almost certainly attribute her condition to this new, pathogenic, *de novo PPP2CA* variant, and not to any other familial, genetic cause. We also did not consider the compound heterozygous *SKIVL2* variants as pathogenic, given the very different clinical image and lack of phenotypic fit with the reported *SKIVL2*-related trichohepatoenteric syndrome (Fabre et al., 2012).

The current case report further highlights that we likely have not yet seen the full clinical and molecular spectrum of the *PPP2CA*-related disorder and that yet more patients with additional new variants might be encountered and diagnosed in the near future, especially at the milder end of the spectrum. Future research should not only keep an eye on such new diagnoses, but should also start focusing on studying the functional implications of *PPP2CA* mutations on brain development and neuronal signaling. For these purposes, the generation of appropriate cell and *in vivo* models of these disorders is eagerly awaited.

## Data Availability

The original contributions presented in the study are included in the article/Supplementary Material; further inquiries can be directed to the corresponding authors.
